# DNA-Based Nanobiosensors as an Emerging Platform for Detection of Disease

**DOI:** 10.3390/s150614539

**Published:** 2015-06-19

**Authors:** Khalid M. Abu-Salah, Mohammed M. Zourob, Fouzi Mouffouk, Salman A. Alrokayan, Manal A. Alaamery, Anees A. Ansari

**Affiliations:** 1 Research Chair for Medical Applications of Nanomaterials, King Saud University, PO Box 2454, Riyadh 11451, Saudi Arabia; E-Mail: salrokayan@ksu.edu.sa; 2 King Abdullah Institute for Nanotechnology, King Saud University, PO Box 2454, Riyadh 11451, Saudi Arabia; E-Mail: aneesaansari@gmail.com; 3 King Abdulla International Medical Research Center, King Abdulaziz Medical City, PO Box 22490, Riyadh 11426, Saudi Arabia; E-Mail: alaameryma@ngha.med.sa; 4 Department of Chemistry, Alfaisal University, Al Zahrawi Street, Al Maather, Al Takhassusi Rd, Riyadh 11533, Saudi Arabia; E-Mail: mzourob@alfaisal.edu; 5 Chemistry Department, Faculty of Science, Kuwait University, PO Box 2969, Kuwait; E-Mail: fmouffouk@ku.edu.kw

**Keywords:** DNA nanobiosensors, nanoparticles, cancer, genetic and infectious diseases, electrochemical and optical sensing

## Abstract

Detection of disease at an early stage is one of the biggest challenges in medicine. Different disciplines of science are working together in this regard. The goal of nanodiagnostics is to provide more accurate tools for earlier diagnosis, to reduce cost and to simplify healthcare delivery of effective and personalized medicine, especially with regard to chronic diseases (e.g., diabetes and cardiovascular diseases) that have high healthcare costs. Up-to-date results suggest that DNA-based nanobiosensors could be used effectively to provide simple, fast, cost-effective, sensitive and specific detection of some genetic, cancer, and infectious diseases. In addition, they could potentially be used as a platform to detect immunodeficiency, and neurological and other diseases. This review examines different types of DNA-based nanobiosensors, the basic principles upon which they are based and their advantages and potential in diagnosis of acute and chronic diseases. We discuss recent trends and applications of new strategies for DNA-based nanobiosensors, and emphasize the challenges in translating basic research to the clinical laboratory.

## 1. Introduction

Deoxyribonucleic acid (DNA) is a molecule found in cells of humans and almost all living organisms. Nearly every cell in the human body contains the same DNA. It comprises information used in our everyday metabolism and physiological activities and influences most of our characteristics. The specific DNA code provides the instructions for each protein to function in a specific manner. Each gene in the DNA encodes a specific protein, which may be involved in different functions and may play a major role in the cell, such as cell signaling and the pathogenesis process of human diseases. DNA is an ideal material for nanofabrication of rigid compositions because its implementation is relatively simple as its assembly can be controlled by base pairing and is comparatively of low cost. The determination of DNA sequences plays an important role in investigating particular diseases. The molecular diagnostics based analyses of genomic sequences have offered a highly sensitive and quantitative method for the detection of infectious diseases, pathogens, and genetic variations. Simple, ultrasensitive detection of sequence-specific DNA has been the aim of many studies and technologies for decades.

Recently, the field of nanotechnology has accelerated the process of integration of various scientific fields, thereby enhancing the breakdown of boundaries between already known disciplines. This eventually led to the emergence of the new interdisciplinary science of nanotechnology and its interrelated biology branch of nanobiotechnology. Advances and developments in this branch culminated in the establishing of nanomedicine, which includes amongst other disciplines, diagnostic materials and devices, molecular imaging, drug delivery systems and regenerative medicine [1]. Remarkably, nanomedicine enables *in vitro* and *in vivo* non-invasive diagnosis and targeted therapy by novel discoveries in sensing, processing, and operating processes. Currently, imaging tools based on nanotechnology have been medically applied as non-invasive methods of diagnosis [2,3,4]. The categories of nanodiagnostic technologies, in addition to DNA-based nanobiosensors, include nanoproteomic-based diagnostics, nanoparticle-based immunoassays, nanoparticulate biolabels, nanoscale visualization (e.g., scanning probe microscopy, scanning electron microscopy), biobarcode assays, nanobiotechnology-based, biochips and microarrays, and combinations of multiple diagnostics technologies.

Nanobiosensors have been increasingly used in medical diagnostics for continuous monitoring of human health [1,2,5,6,7], in addition to their applications in the field of food analysis [8], bioterrorism [9,10], and environment [9,10,11,12]. Presently, the most fascinating and prospective nanobiosensors are DNA-based types [13,14,15,16,17,18,19,20].

Unlike enzymes or antibodies, nucleic acid recognition layers in DNA-based nanobiosensors can be readily prepared and regenerated for multiple use. DNA-based nanobiosensors can be constructed by immobilizing single stranded probes on different electrodes using electro active-indicators to measure the hybridization between DNA strands and their complementary DNA probes [21,22,23]. The detection of specific DNA sequences is significant, not only in clinical diagnostics, but it is also of increasing importance in environment and food analysis [3,24,25]. Here, we focus on the potential of DNA-based nanobiosensors that can selectively target markers of acute and chronic diseases and highlight the challenges in translating some of the basic research to the clinical laboratory.

## 2. What Are DNA-Based Nanobiosensors

**Figure 1:**
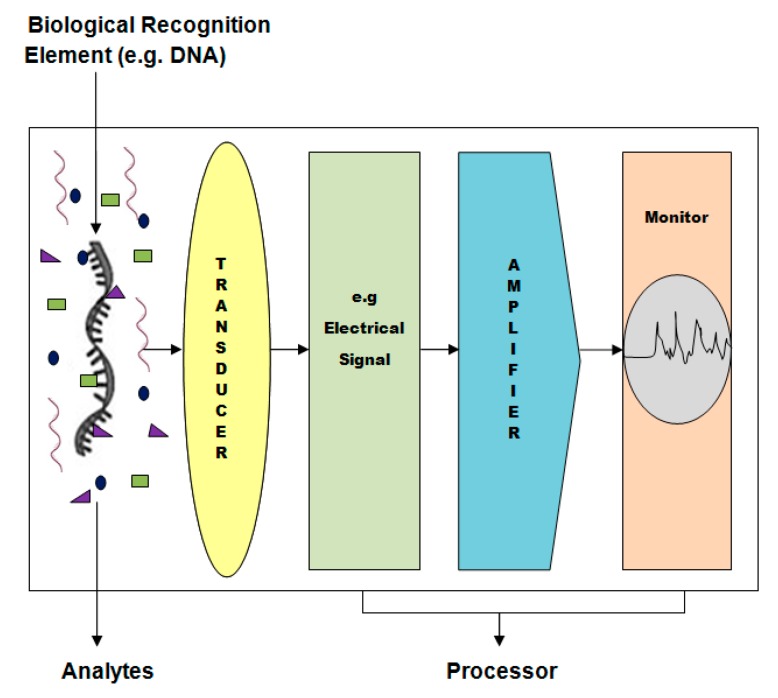
A schematic diagram shows basic biosensor assembly with a biological recognition element, transducer, and processor.

A biosensor is a device that detects, transmits and records the information on a biological analyte. Examples of analytes include nucleic acids (DNA, RNA), proteins such as enzymes, antibodies and antigens, or other biological component such as glucose. A basic biosensor assembly includes a biological recognition element, transducer, and processor (Figure 1).

A biological recognition element can be consolidated to a number of different transducers. The recognition or sensing element, such as nucleic acids, antibodies, enzymes, proteins, or whole cells, can be integrated with a transducer via immobilization by covalent interaction, cross-linking, or adsorption. The transducer converts the molecular biological signal into a digital or electric signal proportional to the concentration of analyte and can be amplified, quantified, displayed, and analyzed through a processor. Nanostructured materials based transducers enhance the sensitivity by more than one order of magnitude compared to that observed at nanomaterials-bare, such as high loading of recognition element and a better electrical communication ability of the nanomaterials conventional electrodes [5,6,26,27]. This improved analytic performance is attributed to factors such as better electrical communication ability of nanomaterials (nanotubes, nanorods, and nanofibers) and high loading of recognition elements. The transducer function depends on the parameter to be measured. It may be optical (measurement of colorimetric, fluorescent, luminescent, and interferometric changes), potentiometric (potential measurement at constant current) [28], amperometric (current measurement at constant potential) [29], piezoelectric and acoustic waves (measurement change in mass) [30], or calorimetric (measurement changes in temperature) [31].

**Figure 2:**
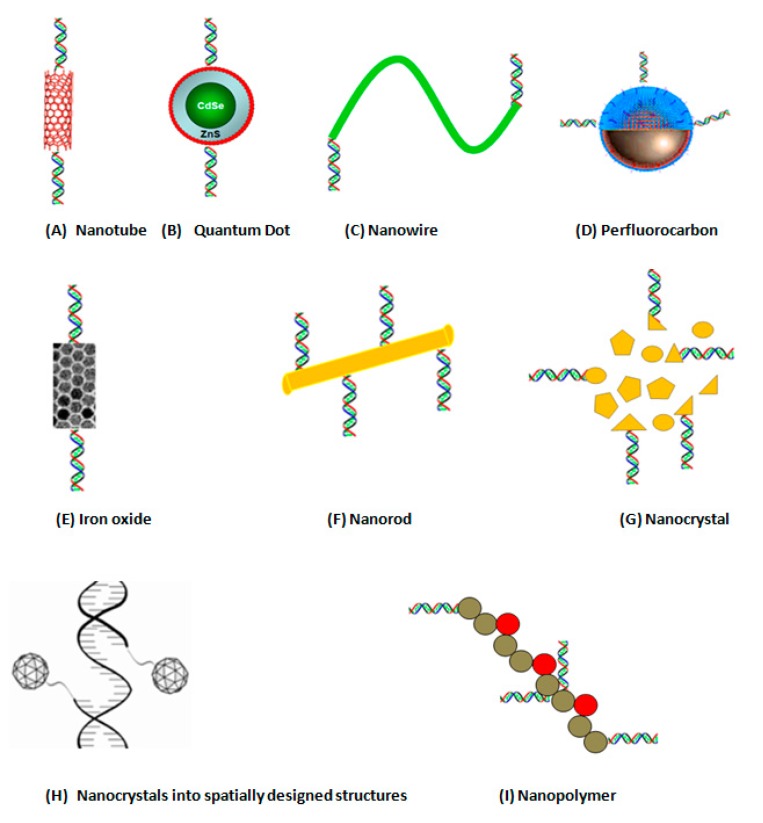
Different types of nanostructures-based transducers to which DNA can be attached.

The above functional types of biosensors may make use of nanomaterials as transducing substances. These nanomaterials include: Semionductor nanoparticles, or so called quantum dots (QD), silver, silica, perfluorocarbon and organic polymers, surface enhanced Raman spectroscopy (SERS) nanotags, and fluorescent lanthanide nanorods (Figure 2).

A nanobiosensor is a biosensor that acts at the nano-metric level on a nanoscale region. Several types of nanobiosensors have been proposed for use in clinical and laboratory diagnosis. These types include optical, electrochemical, electrical and electronic, nanowire, nanotube-based, viral, nano-shell, and quartz nanobiosensors. DNA-based nanobiosensors, which involve nucleic acid recognition processes, are rapidly being used for assay development towards simple and rapid testing of genetic material and infectious agents. DNA-based nanobiosensors, including gene chips, are of major interest due to their tremendous promise for detection of disease and obtaining sequence—specific information in a faster, simpler and cost-effective manner compared to the traditional hybridization techniques [32,33]. The DNA probe is either chemically or enzymatically labeled with chemiluminiscent or radioactive probes or ligands, such as biotin, since the unlabeled nucleic acid is not able to provide any signal by itself.

## 3. DNA-Based Nanobiosensors for Detection of Infectious Diseases

### 3.1. Optical Nanobiosensors

The rapid and sensitive detection of pathogenic microorganism at the point of care is essential in disease management and in impairing health outcomes. Most of the conventional microbiological diagnostic methods lack ultra-sensitivity and are limited by a delay in getting results. The most common DNA-based nanobiosensors make use of fluorescent nanostructures. Fluorescence occurs when a valence electron is excited from its ground state to an excited state. An electron emits a photon when it returns to its original ground state. Biosensors based on fluorescence resonance energy transfer (FRET) technology, which are used in probe-based assays for quantitative PCR (qPCR), make use of the transfer of energy from a donor fluorophore to an acceptor fluorophore when appreciable overlap exists between the absorption spectrum of the acceptor and the emission spectrum of the donor. This technology has also been used in screening gene mutations, measuring gene expression, and in quantifying viral loads [34,35,36,37].

**Figure 3:**
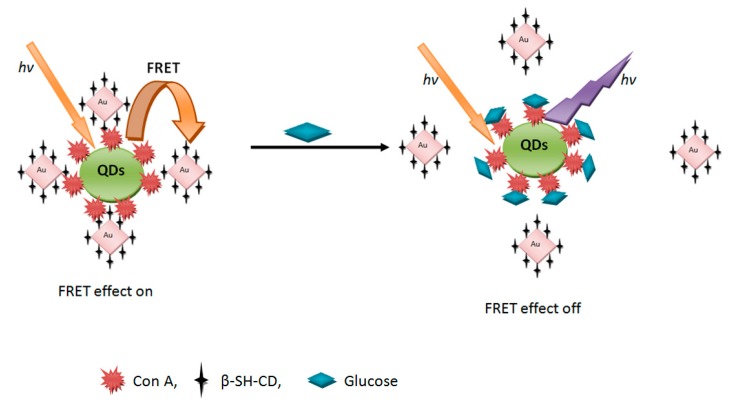
Chemical structure of the QDs-ConA-β-CDs-AuNPs nanobiosensor and schematic illustration of its FRET-based operating principles.

Significant works have been reported in the literature on the utilization of nanostructured materials for construction of fluorescence sensors. In this context, semiconductors QDs have been used for proteins and DNA sensing. Melvin *et al.* [36] developed a fluorescence competition assay for DNA detection using gold nanoparticles (NPs) and QD as a FRET donor–acceptor couple. The gold nanoparticles are released from the QDs in the presence of complementary oligonucleotides, thus regenerating the fluorescence of the QD. Figure 3 schematically illustrates how this kind of biosensors works.

The detection of single-molecule hybridization has been accomplished by a hybridization-detection method using multicolor single stranded DNA-functionalized QDs as a nanobiosensor [38]. Independent hybridization reaction in the presence of various target sequences leads to the generation of discernible sequence-specific spectral codings. Simultaneous detection of multiple relevant sequences can be used for genetic analysis of anthrax bacteria. Moreover, luminal functionalized silver nanoparticles have been utilized by He *et al.* [39] for ultrasensitive detection of *Mycobacterium tuberculosis* DNA by calculating chemiluminescence activity. On the other hand, a spectroscopic assay, based on SERS using silver nanorods, has been used for rapid, sensitive, and specific detection of viruses [40]. The technique is based on measuring the change in frequency of near infrared laser as it scatters viral DNA or RNA. Spectral differences, based on frequency, between viruses, viral strains, and viruses with gene deletions can be recorded rapidly (≤60 s) without viral manipulation.

### 3.2. Label-Based Electrochemical Nanobiosensors

Fluorimetric and other optical detection methods usually require highly transparent sample solution as the color and/or the fluorescence of the solution might interfere with the fluorimetric or optical signal generated from sensors. Electrochemical detection provides another useful technique for non-transparent samples, such as blood and urine, since the signal transduction can be achieved by non-fluorimetric or optical means [41]. Electrochemical devices received considerable attention in the development of sequence-specific DNA hybridization biosensors [42,43]. These devices offer smart routes for relating the DNA recognition element to the signal-transduction processes. Biosensors based on DNA hybridization depend on the generation of an electrical signal from the DNA base-pair recognition event [44,45,46]. Such a hybridization process is commonly detected via increased current signal of an electroactive indicator.

### 3.3. Label-Free Electrochemical Nanobiosensors

**Figure 4:**
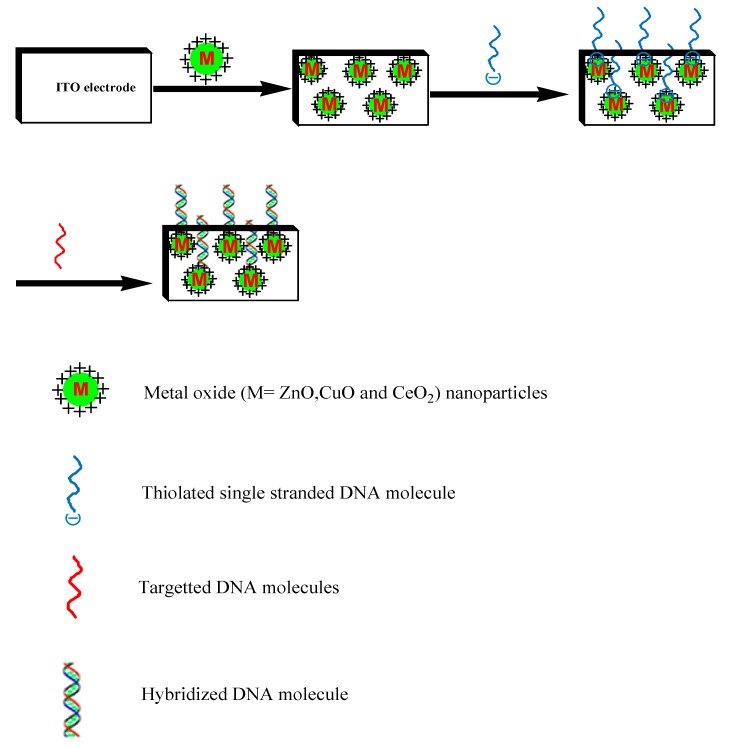
Schematic presentation of immobilization of thiolated single-stranded probe DNA on the surface of ZnO for hybridization detection in double-stranded DNA (target DNA).

Increased interest has grown recently in direct, label-free electrochemical detection systems. Such direct detection can be accomplished by monitoring electrical changes resulting from the hybridization event. In addition the safety is improved by eliminating the need of using an indicator [47,48,49]. Wang *et al.* [50] introduced the first indicator-free scheme. Ansari *et al.* [51] employed a probe specific to *Neisseria gonorrhea*, which is composed of 20-m thiolated oligonucleotide (th-ssDNA) immobilized onto a sol-gel derived nanostructured zinc oxide (ZnO) film, dip-coated onto a glass substrate of indium-tin-oxide (ITO) (Figure 4).

This fabricated DNA nanobiosensor has been used for the detection of the sexually transmitted disease, gonorrhea. The results of the characterization studies performed on this th-ssDNA-ZnO/ITO bioelectrode using X-ray diffraction, scanning electron microscopy, Fourier-transform infrared, UV-visible revealed the linearity as 0.524 amol–0.524 nmol with a detection limit of 0.704 amol within 60 s. Another novel type of electrochemical DNA sensors has been fabricated by immobilizing thiolated single-stranded oligonucleotide (ss-DNA) probe onto gold (Au) coated glass electrode. Nisseria meningitides detection [52] using this type of DNA biosensor was accomplished by hybridization with complementary DNA (Ctr A) in presence of methylene blue (MB). Complimentary DNA in the range of 4–42 ng/mL (hybridization with response time 60 s) could be detected by this type of DNA/Au electrode. The electrode was found to be stable for about four months when stored at 4 °C. The sensitivity of double-stranded (ds-DNA)/Au electrode is 115.8 µA/ng.

A different approach has been used by Singh *et al.* [53] to fabricate a nanobiosensor for detection of *N. gonorrhea*. A nanobiosensor based on a multi-copy gene of *N. gonorrhea* functionalized nanostructured–polyaniline coated onto ITO-coated-glass plate has been fabricated. Avidin-biotin was used as a cross-linking agent in this type of biosensors. This DNA functionalized electrode can specifically detect up to 0.5 × 10^−5^ M of complementary target within 60 s of hybridization time at 25 °C by differential pulsed voltammetry (DPV) using methylene blue as electro-active DNA hybridization indicator. Moreover, the fabricated nanobiosensor can distinguish presence of *N. gonorrhea* from *N. meningitides* and *Escherichia coli* in cultures from urethral swabs of patients. Recently, Singh *et al.* [54] reported the use of a chitosan/Fe_3_O_4_ nanocomposite dispersion deposited on ITO electrode as a new platform for detection of gonorrhea. Chitosan is a biopolymer and has been used as an effective dispersant of nanoparticles for film fabrication [55]. The nanocomposite films deposited on electrode surface were mechanically stable and a further stabilization of the chitosan film containing iron oxide nanoparticles, achieved by chemical cross-linking with free aldehyde groups and avidin-biotin coupling, produced a substrate used for covalent immobilization of ssDNA and rapid and specific (mismatch-discriminating) DNA hybridization detection. The detection of oxygen uptake by chitosan (CH)-iron oxide (Fe_3_O_4_) nanocomposite via immobilization of biotinylated probe DNA was robust and applied for assays. A low detection limit (1 × 10^−15^ M) and very high assay throughput rate (1 × 10^−16^ M to 1 × 10^−6^ M) was achieved. Other analytic applications of chitosan doped nanomaterials have been reported in the literature, such as a biosensor based on chitosan doped with multiwall carbon nanotube (MWCNT), have been successfully used to detect gonorrhea. Methylene blue was employed as a DNA indicator. It was found that carbon nanotubes (CNTs) can enhance the electroactive surface area three fold (0.093 + 0.060 and 0.28 + 0.03 cm^2^ for chitosan-modified electrodes and chitosan-CNT, respectively). The resulting DNA biosensor shows a wide concentration range from 1 × 10^−6^ M to 1 × 10^−17^ M with a detection limit of 1 × 10^−16^ M. The amperometric response time is 60 s and extremely stable for about four months when stored at 4 °C [56]. In another approach Singh *et al.* have applied polyaniline/carbon nanotubes hybrid nanocomposite for immobilization of 5′-amino-labeled *N. gonorrhea* probe using glutaraldehyde as a cross-linker for sensitive and selective detection of gonorrhoea. The biosensor exhibits excellent response performance to gonorrhea with the linear range from 1 × 10^−6^ M to 1 × 10^−7^ M and a detection limit of 1.2 × 10^−17^ M. Furthermore, the biosensor shows high sensitivity, rapid response, long-term stability, good reproducibility and freedom of interference from other coexisting electroactive species [57].

Electrochemical sensors have also proved to be very useful for detecting small DNA damages induced by various enzymatic digestion, chemical agents, or ionizing radiation [58]. Such electrical detection is strongly dependent on the DNA structure. In addition to monitoring changes in tensammetric signals of DNA, such studies examine the response of electroactive damaging agents, such as carcinogens interacting with DNA in connection to the use of DNA-modified electrodes. Anticancer drugs in body fluids could be biosensed with similar electrodes [59]. Another approach which could be of potential applications in detecting viral markers demonstrated the use of DNA as a template for the fabrication of polyaniline nanowires [60]. Oriented DNA strands were formed on a thermally oxidized Si surface, then incubated in a solution of aniline monomer. The aligned aniline monomers were polymerized enzymatically to produce the nanofibers. The sensors were constructed by immobilizing DNA strands across an array of gold electrodes. A variation of electrical conductivity of polyaniline (PANI) nanofiber approach was recently reported by Fan *et al.* [61] to detect micro RNA (miRNA). To achieve this, capture probes of peptide nucleic acids (PNA, with sequence complementary to the miRNA target, were immobilized in the gaps of microelectrodes array. Addition of horseradish peroxidase, aniline, and H_2_O_2_ resulted in the formation of polyaniline nanofibers only when miRNA was hybridized to the PNA target. The amount of miRNA present was found to correlate to the conductance of the deposited PANI. The above technique could be potentially developed to detect miRNA specific for some pathogenic bacteria and viruses.

Malhotra and his coworkers constructed a sensitive amperometric nucleic acid biosensor wherein 21-m oligonucleotide probe (ss-DNA) is immobilized on nano-structured zirconium oxide (ZrO_2_) film deposited onto gold electrode for specific detection of Mycobacterium tuberculosis [62]. The resulting biosensor based on nanostructured metal oxide films display excellent electrocatalytic responses, which can be used for early and rapid diagnosis of *M. tuberculosis* with a detection limit of 0.065 ng/μL within 60 s. Zirconia nanoparticles grafted with chitosan (CHIT) (considering that chitosan, with abundant amino groups, exhibits excellent film-forming ability for the solubility in slightly acidic solution due to the protonation and insolubility in solution with pH above pKa (6.3) for deprotonation) has been applied as matrix to covalently immobilize the ss-DNA probe selective for *M. tuberculosis* detection. Chitosan improved the biocompatibility, toxicity, thermal, and chemical stability of the nanocomposite and enhanced the electron transfer ability efficiently between the analyte and the electrode surface [63]. Cyclic and differential pulse voltammetry measurements were used to investigate the electrochemical response of DNA/CHIT-NanoZrO_2_/ITO bioelectrode. The sensitivity of the bioelectrode observed was 6.38 × 10^−6^ AµM^−1^ could detect the complementary target DNA up to 0.00078 μM. In another approach similar group employed zirconia nanoparticles grafted MWCNTs based electrochemical impedometric biosensor for *M. Tuberculosis* detection [64]. The CNTs can amplify protein or DNA recognition and transduction events, which could be utilized as an ultrasensitive method for electrical biosensing of DNA. The carboxylic groups of MWCNTs of the electrode were covalently linked to the oligonucleotides with amine groups at the 5′ end. The hybridization events were monitored by electrochemical impedance spectroscopy measurements of the intercalated DNA of Nisseria gonorrhea. The ability of carbon nanotubes to allow electron-transfer reactions, the high surface area and catalytic activity of zirconia nanoparticles and the sensitivity of developed electrochemical DNA biosensors have been significantly improved. The bioelectrode could selectively detect target DNA concentration ranging from 1 × 10^−2^ to 1 × 10^−8^ mM with a superior detection limit of 0.01 nM.

Tam *et al.* [65] have managed to covalently immobilize probe DNA on MWCNTs for label free and direct detection of influenza virus (type A). The investigators used Raman and FTIR spectra for the establishing of covalent bonding in between phosphate groups and amines of the DNA sequence. Target DNA down to 0.5 nM coud be detected by the fabricated DNA biosensor [65]. Recently, semiconductor (quantum dot, QD), e.g., CdSe, CdS, PbS and ZnS nanoparticles have attracted scientists in electrochemical biosensor applications. Owing to their unique (size-tunable fluorescent) properties they cause the labels in the electrochemical biosensors to be very sensitive [66]. Wang’s group was the first to prove the concept by using semiconductor nanoparticle labels for the assay of electrochemical DNA hybridization, and then it was extended to electrochemical DNA biosensors and immunosensors [67,68]. Fan *et al.* reported an electrochemical DNA biosensor for the measurements of avian influenza virus based on the use of semiconductor quantum dots [66]. A label-free or indicator-free DNA hybridization detection has been achieved by electrochemical signal measurements based on ss-DNA/CdSe modified GCE. The present method has different combining ability of ss-DNA and ds-DNA with CdSe nanostructures as measured by the change of electrochemical signal. The proposed biosensor based on quantum dots reveals excellent sensitivity and selectivity for detecting the Avian Influenza Virus (AIV) DNA sequences.

Potentiometric biosensors were developed by conjugating carbon nanotubes with aptamers [69]. This technique allowed label-free detection of *Staphylococcus aureus* in real time and promises to allow the detection of other types of bacteria in a similar manner. In this technique anti-*S. aureusa* aptamers are the recognition element and a network of single-walled carbon nanotubes (SWCNTs) acts as an ion-to-electron potentiometric transducer. Carbon nanotubes were functionalized with aptamers either covalently or non-covalently. The minimum concentration that could be detected with covalent modification was 8 × 10^2^ colony forming units (CFU)/mL and the sensitivity was 0.36 mV/decade. With the non-covalent approach, the minimum concentration detected was significantly affected (10^7^ CFU/mL) but the sensitivity was higher (1.52 mV/decade). Nevertheless, in both cases, the potential demonstrated a linear mode as a function of decade of bacteria.

### 3.4. DNA-Based Piezoelectric Biosensors

A piezoelectric DNA-based biosensor was developed for the direct detection of Mycobacterium Tuberculosis (MTB) in clinical specimens [70]. The technique involved immobilization of specific synthetic biotinylated probe that was designed from IS6110 gene-specific for MTB [71,72] to the surface of a quartz crystal. Detection of the target DNA was achieved by measuring the frequency change of the quartz crystal occurring upon hybridization of the target DNA with DNA biotinylated probe. Concentrations of non-amplified genomic bacterial DNA-target as low as 0.5 µg/mL could be detected. The sensitivity of this technique could be improved and its applications could be diversified by designing DNA biotinylated probes that suit different DNA targets from different pathogenic microorganisms.

## 4. Diagnosis of Genetic Diseases with DNA-Based Nanobiosensors

The study of gene polymorphism by analyzing gene sequence play a crucial role in rapid detection of genetic variations allowing for the potential of fairly reliable diagnosis, even before any symptoms of a disease appear. Many detection techniques have been developed that rely upon target hybridization with fluorescent [73,74,75], radioactive, chemiluminescent [71], or other types of labeled probes [76,77]. Additionally, there are indirect detection methods that are based on enzymes, which catalyze the generation of colorimetric, chemiluminescent, or fluorescent signals [22,78].

Maxwell *et al.* [73] developed a unique method for exploring single base mutations and specific DNA sequences. Their assay relied on labeling oligonucleotide molecules with a thiol group which were attached at one end of a 2.5 nm gold nanoparticle and a fluorophore at the other end. The conformation opens upon target binding and the fluorophore leaves the surface allowing for fluorescence to be consequently restored. Thus, a fluorescent signal is generated as a result of this structural change. The signal was found to be specific and highly sensitive to the target DNA [74].

The molecular beacon (MB), a polynucleotide molecule, is another fairly recent fluorescent probe. It provides exceptional advantages for gene analysis [74]. MBs are designed to form a stem loop structure with a quencher to 3′ end and a fluorophore linked to the 5′ end. The close proximity of the quencher and the fluorophore does not allow for the appearance of any fluorescence. Upon hybridization of the target oligonucleotide with the MB, the quencher and fluorophore are spatially separated, causing the fluorescence signal to be restored. The above two techniques could be adapted to detect changes in specific DNA sequences and single base mutations in a variety of genetic diseases.

Optical fluorometric biosensors have been gaining increasing importance due to their high sensitivity and simplicity [73,74]. Their potential to detect multiple genes on DNA biochips allows for rapid and multiplex analysis of nucleic acid samples, including detection of infectious agents [68], diagnosis of genetic diseases [73], measurements of differential gene expression [79], forensic analysis, and drug screening [80,81,82,83,84].

An obvious challenge in the area of DNA detection is the development of methods that do not rely on target amplification systems such as polymerase chain reaction. One remarkable example is the utilization of nanowires as nanobiosensors, to differentiate between mutant and wild type and genes, for transmembrane receptor protein of cystic fibrosis. The change in chemical potential resulting from a target/analyte binding event, such as DNA hybridization constitute the basis for nanowire operation [85]. A field effect transistor operates by a similar effect. For example, 20-nm-wide silicon nanowires were grown onto catalytic nanoparticles using the technique of vapor deposition [85]. Groups of peptide nucleic acid (PNA) bound by biotin linkers to avidin proteins consisted the molecular elements of recognition on the nanowire. The full sequence of the gene for the cystic fibrosis transmembrane receptor protein was contained in the PNA probes. Thus they were able to bind wild type DNA sequences leading to an abrupt two fold increase in conductance. The authors proposed that this was consistent with an increase in negative surface charge density resulting from binding of negatively charged DNA, at the surface of semiconducting nanowire. Nevertheless, exposure of the device to mutant F 508 DNA also led to a similar change in conductance due to nonspecific binding of the DNA. However, mutant DNA could be easily removed from nanowire surface by washing with DNA free solution, while wild type DNA became firmly bound to the PNA receptors and could not be removed. One main advantage of nanowire sensors is that the density and number of the sensor elements is restricted only by the ability to electronically suit individual nanowires. Thus, measurements of large numbers of different genes and proteins from single cells or very small tissue samples can be achieved upon constructing large scale circuits within very small environment [86]. Park *et al.* advanced another example, which utilizes conductivity changes in DNA detection without target amplification [77]. They reported a DNA array detection method in which the binding of oligonucleotides functionalized with gold nanoparticles led to conductivity changes associated with target-probe binding events. Silver deposition bridges the electrode gaps in which gold nanoparticles were localized and led to readily measurable conductivity changes. Using this method target DNA at concentrations as low as 500 femtomolar with a point mutation selectivity factor of about 100,000:1 could be detected.

Nanoparticle amplified surface plasmon resonance has been utilized for ultrasensitive detection of DNA hybridization. Use of the Au nanoparticle tags led to a more than 100 fold improvement in sensitivity for the target oligonucleotide as compared to the unamplified binding event [87].

This new approach demonstrated that the oligonucleotide linked quantum dots (semiconductor nanocrystals) could soon become useful biosensors in molecular biology for varied applications, such as detecting mutations and mapping of genes. The specific interaction between complementary nucleic acid strands could be monitored either by the formation of nanocrystal aggregates [88,89] or via the sorting of differently colored nanocrystals in media supporting the matching oligonucleotides [90].

Other types of biosensors made use of conjugates of self-assembled DNA-streptavidin. For example, the conjugates were used as calibration standards of ion switchable nanoparticle networks for scanning probe microscopy [91,92], which could be used for sensitive scanning of a large variety of pathological specimens. The ultimate power of integrating bionanotechnology into complex DNA-based biological systems will emerge as a revolutionary tool for ultrasensitive detection of disease markers and infectious agents. Such detections are necessary for medical diagnosis and fight against bioterrorism.

## 5. Applications of DNA-Based Nanobiosensors in Management of Cancer

DNA hybridization detection has attracted considerable interest for its wide applications in cancer and genetic diseases diagnosis. Various techniques have been devised for DNA hybridization detection. These include electrochemical [93], fluorescence [94], enzymatic [95], surface plasmon resonance spectroscopy [96], quartz crystal nanobalance and colorimetric [78,97,98] techniques. Electrochemical transducers, for example, are often being used due to their low cost, simplicity, small dimensions, high sensitivity and compatibility with micro fabrication technology [99,100,101].

Many methods including adsorption [102], self-assembly [103,104], covalent binding [105,106], biotin -avidin interactions [107] and entrapment in a polymer matrix [108] have been employed for the surface-immobilization of ss-DNA probes on electrodes. Some of these methods are relatively expensive and complex with sensitivity below pmol·L^−1^ level. Additionally, the activity of ss-DNA is not retained in some of them.

Ceramic oxides have attracted increasing attention and have been successfully employed in DNA immobilization and its subsequent hybridization with the target. Fabricating DNA hybridization biosensors could be achieved, for example, by applying electro-deposited ZnO_2_ thin films on gold electrodes [109]. The linear range of detection obtained, however, was very narrow. Other investigators, such as Liu *et al.* [110], reported DNA immobilization based on ZnO_2_ gel. The brittleness of such matrix, however, limited its practical application. To overcome some of the limitations of ceramic materials, they have been combined with organic materials to be used as composite for ss-DNA immobilization. For example, Feng *et al.* [111] reported the fabrication of a nano-porous CeO_2_/chitosan composite film as the immobilization matrix for colorectal cancer DNA sequence. Such matrix combined the excellent film forming ability of chitosan as a natural cationic polymer and the affinity of nano-CeO_2_ towards the oxygen of DNA in addition to its good biocompatibility, nontoxicity and electronic conductivity. After hybridization reaction differential pulse voltammetry (DPV) was used to record the signal response of methylene blue (MB) which demonstrates different affinity for ss-DNA and ds-DNA. This response was used to detect and determine the amount of colorectal cancer target DNA sequence. The established electrochemical biosensor has a relatively wide detection range from 1.59 × 10^−11^ to 1.16 × 10^−7^ M and the ability to discriminate completely complementary target sequence and four-base-mismatch sequence.

Wang *et al.* [112] reported a different attractive approach which involves the attachments of biotinylated oligonucleotide probes onto streptavidine-coated magnetic beads, followed by the dissociation of the DNA hybrid from the beads and potentiometric stripping measurement at a renewable graphite pencil electrode. This protocol was applied successfully to the assay and quantitation of DNA sequences related to the breast-cancer BRCA1 gene.

**Figure 5:**
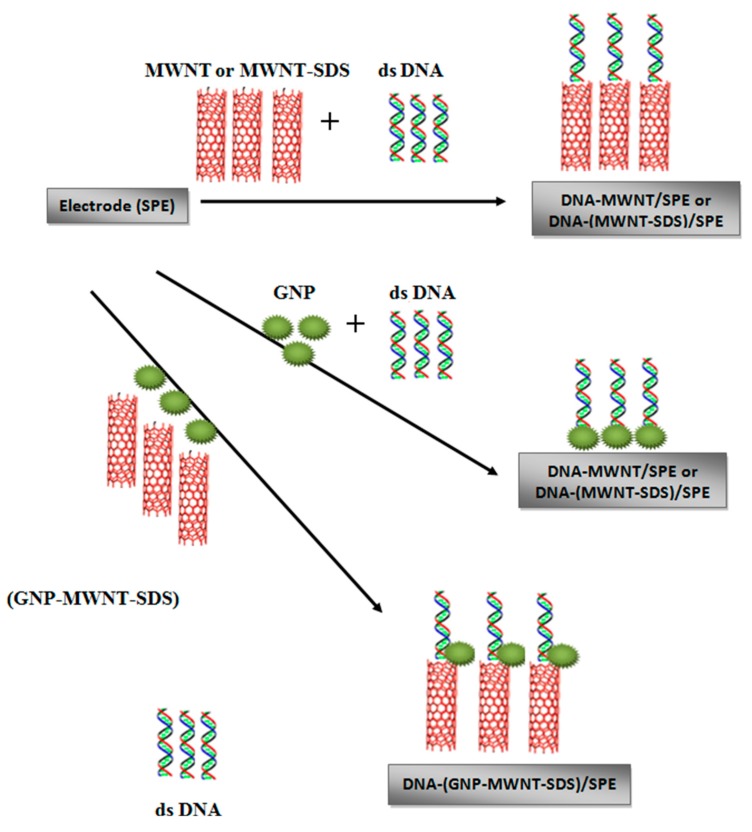
Schematic illustration of preparation of the nanostructured film of mixed (DNA-nanomaterial/SPE) [113].

A novel electrochemical biosensor for berberine monitoring based on MWCNTs immobilized with ssDNA on screen-printed carbon electrodes (SPE) has been reported by Ovadecova *et al.* [113]. The DNA-(GNP–MWNT-SDS)/SPE electrode has been used for detection of effect of berberine on DNA from cancer cells. The electrode was prepared by dispersion of MWCNTs in dimethylformamide (DMF) or a colloidal gold nanoparticles (GNP) in phosphate buffer solution (PBS), solution of sodium dodecyl sulfate (SDS), and a DNA aqueous solution followed by casting of the prepared ssDNA/MWCNTs on the electrode (Figure 5). Ovadecova *et al.* [113] have also shown the synergy effect between CNTs and redox marker Co[(phen)_3_]^3+^ with the significant improvement of redox activity of guanine moiety. The obtained results for DNA-(GNP–MWNT-SDS)/SPE demostrated a 104-fold increase in the sensitivity toward berberine compared with only DNA modified electrodes. Based on this result, the authors contributed the observed remarkable enhancement in the detection sensitivity to the significant increase in the surface area of the electrode caused by the presence of DNA-(GNP–MWNT-SDS)/SPE in the composite, thus allowing a higher density of boronic acid groups to be available for dopamine binding. The resulting biosensor is effective for the detection of berberine effect on human cancer cell line (U937), which had a very strong effect on the structural stability of DNA at relatively low concentrations. Non-cancer cells were affected only at relatively high concentrations of berberine (75 and 50 μg·mL^−1^) [113].

The detection of prostate specific antigen (PSA), as an indicator of breast cancer and prostate cancer were amplified using gold nanoparticles. To achieve this, magnetic particles (MP) were immobilized with a primary antibody targeting PSA, and gold nanoparticles were activated by both a secondary antibody for the PSA and numerous copies of a specific non-human short DNA duplex through end linking on the sense strand of the duplex. After the primary antibody-antigen reaction, MPs were concentrated and washed at a specific location by applying a magnetic field. These MPs were then reacted with activated gold nanoparticles and subjected to several washes under a magnetic field to remove excess nanoparticles. The antisense strands of the specific DNA duplex were released into solution by heat denaturing the MP-nanoparticle duplex. The mount of oligonucleotide released was assayed using a DNA microchip format before the PCR. A detection sensitivity of PSA at a concentration as low as 30 aM could be achieved. By using the same approach, a concentration down to 500 zM (10^−21^ M) could be obtained [114].

A new bio-barcode amplification (BCA) method was developed that allows detection of molecules at low attomolar levels without the need for PCR amplification [114,115,116]. The target protein to be detected was ‘sandwiched’ between two probes: a targeting probe and a capture probe. Both probes had antibodies that could bind to the protein to be targeted. The resulting complexes were separated from solution using a magnetic field and washed till the tags were denatured from their complements. Geoorganopoulou *et al.* [117] used scanometric DNA detection for the final product obtained in the BCA technique. In their technique, tag DNA was captured on a glass slide, which was already spotted with DNA strands that were complementary to part of the tag. The nanopaticles were coated with silver followed by measuring the light intensity scattered from each spot. BCA has been successfully used for the detection of PSA [115,116] and could be successfully applied to other types of cancer markers.

Other newly developed DNA-based nanobiosensors could have the potential to detect different cancer markers. One such biosensor takes advantage of the redox state change induced by catalysts immobilized on DNA aptamers [118,119]. This electrochemical biosensor was designed for the detection of thrombin and was based on the redox change induced by platinum nanoparticles (Pt NPs) immobilized on DNA thrombin aptamer. The Pt NPs catalyzed H_2_O_2_ reduction to H_2_O results in cathodic current, enabling thrombin detection with a detection limit as low as 1 nm. To extend the methodology to multiplexed detection, Wang and co-workers [120] used protein labeled quantum dots (QDs) with different chemical compositions. Aptamers for thrombin and lysozyme, for example, were immobilized on Au surface PbS labelled lysozyme and CdS labelled thrombin were then bound on the aptamers, respectively. In the presence of protein analyte, the protein replaced corresponding QD labelled protein on the surface. Electrochemical stripping detection was employed to monitor the remaining QDs and hence the amount of protein in sample solution. The same technology could be extended to other types of proteins especially cancer markers such as Ca 15-3, 27, 125, bladder tumor antigen (BTA), carcinoembryonic antigen (CEA), alpha-fetoprotein (AFP), IL-10, as well as small molecules, as long as the required suitable aptamers are made available. Aptamers-free DNA sensors, however, for detection of bladder cancer biomarkers in urine has been reported recently [121]. In this study, a label-free DNA optical sensor based on silicon microring resonators has been developed. This approach will provide hopefully a highly sensitive and specific platform for genetic analysis in cancer diagnostic and surveillance.

## 6. DNA-Based Nanobiosensors for Detecting Markers of Immunodeficiency Related Diseases

DNA-based biosensors may assist in identifying different markers of immune deficiency related diseases [122], and may also allow identifying allergens and pollutants [123]. Bio-barcode amplification, for example, has been successfully applied for the detection of the cytokine protein, interleukin that is involved in inflammation in humans [124].

Noorbakhsh *et al.* applied nickel oxide nanoparticles mixed with [Ru(NH_3_)_5_Cl]PF_6_ complex modified glassy carbon electrode to immobilize ssDNA probe used for determination of the complementary probe, taxon: 32630 (TNF) [125]. The result of electrochemical measurements revealed good sensitivity and selectivity for the complementary probe, taxon: 32630 TNF. It had a linear dynamic range, sensitivity and a detection limit from 4 × 10^−10^ M to 1 × 10^−8^ M, 34.32 nA.nM^−1^ and 6.8 × 10^−11^ M, respectively. The proposed biosensor exhibited excellent reproducibility and stability with quite simple and relatively low-cost preparation.

A colorimetric bio-barcode method that minimizes the requirements of conventional nonenzymatic cytokine detection assays has been reported by Nam *et al.* [124]. Their method relied on porous microparticles, which enabled loading of a large number of barcode DNA per particle and gold nanoparticles-based colorimetric barcode detection. The sensitivity of this method goes up to detecting 30 aM concentrations of cytokines (approximately three orders of magnitude more sensitive than other non-enzymic assays). This assay expected to be very useful in diagnosing immunological disorders which involve cytokine(s) as marker(s) [124].

An increase in the sensitivity of TNF-α detection was also reported by using immunopolymerase reaction technique (IPCR) [126]. IPCR is based on chimeric conjugates of nucleic acid molecules and specific antibodies, the former of which are used as markers for signal generation after their PCR amplification. Table 1 summarizes the characterized parameters of some of the published literature reports on DNA biosensors.

**Table 1:** Characterized parameters of some of the published literature reports on DNA biosensors.

Immobilization Matrix	Detected Microorganisms/Protein/Virus	Linearity	Detection Limit	Shelf Life	Sensitivity	Response	Reference
**Ag nanoparticles**	*M. tuberculosis*	0.1–7.0 fM	0.03 f M	-	-	-	He *et al.* 2011 [39]
**ZnO nanoparticles**	*Neisseria gonorrhea*	0.000524 fmol–0.524 nmol	0.000704 fmol	-	-	60 s	Ansari *et al.* 2009 [51]
**Au electrode**	*Nisseria meningitides*	7–42 ng/mL	-	4 months	115.8 µA/ng	60 s	Patel and Malhotra, 2010 [52]
**PANI nanoparticles**	*Neisseria gonorrhea*	1 × 10^−^^16^ to 1 × 10^−^^6^ M	0.5 × 10^−15^ M	-	-	60 s	Singh *et al.* 2009 [53]
**Chitosan-iron oxide nano-composite**	*Neisseria gonorrhea*	1 × 10^−16^ M to 1 × 10^−6^ M	1 × 10^−15^ M	4 months	-	60 s	Singh *et al.* 2011 [54]
**Chitosan-MWCNT**	*Neisseria gonorrhea*	1 × 10^−6^ M to 1 × 10^−17^ M	1 × 10^−16^ M	4 months	-	60 s	Singh *et al.* 2010 [56]
**Polyaniline/carbon nanotubes**	*Neisseria gonorrhea*	1 × 10^−6^ M to 1T10S17 M	1.2 × 10^−17^ M	75 days	-	60 s	Singh *et al.* 2010 [57]
**ZrO_2_**	*Mycobacterium tuberculosis*	640–0.065 ng/μL	0.065 ng/μL	4 months	7.9 × 10^−7^μL/ng	60 s	Das *et al.* 2010 [62]
**Chitosan-ZrO_2_**	*Mycobacterium tuberculosis*	-	0.00078 μM	18 weeks	6.38 × 10^−6^ AμM^−1^	60 s	Das *et al.* 2011 [63]
**ZrO_2_-MWCNT**	Mycobacterium tuberculosis	1 × 10^−2^ to 1 × 10^−8^ mM	0.01 nM	-	-	-	Das *et al.* 2011 [64]
**MWCNT**	influenza virus	-	0.5 nM	-	-	20 min	Tam *et al.* 2009 [65]
**NiO nanoparticles**	taxon: 32630	4 × 10^−10^ M to 1 × 10^−8^ M	68 pM	-	34.32 nA nM^−1^	-	Noorbakhsh *et al.* 2011 [126]

## 7. DNA-Based Nanobiosensors for Diagnosis of Neurological Diseases

The ultrasensitive bio-barcode amplification (BCA) mentioned above, has been successfully applied for the detection of amyloid-B-derived diffusible ligands (ADDLs), which are potential soluble pathogenic Alzheimer’s diseases markers. ADDL concentrations for the subjects diagnosed with AD were found to be consistently higher than the levels in the cerebrospinal fluid taken from non-demented age-matched controls [117].

## 8. DNA-Based Cellular Bioimaging

Early diagnosis and accurate treatment of diseases in general depends on sensitive rapid, and selective detection of diseased cells. Recent diagnosis of cancers such as leukaemia relies on histology and flow cytometry using single dye-labelled antibodies which may not lead to high signal output. To enhance the detection potential of conventional flow cytometry, Esteves *et al.* [127] combined the selectivity of aptamers with ease of surface functionalization offered by dye-doped silica nanoparticles. By using nanoparticles that trap thousands of dye molecules in silica matrix, one or two orders of magnitude can be achieved. Moreover, the ability to prepare dye-doped nanoparticles with almost any of the currently available fluorophores, or even several dyes inside one nanoparticle [128] permits multiplex detection of several analytes simultaneously.

Aptamers like antibodies can bind with high affinity and specificity to a broad range of targets and when combined with nanostructured material can be used to develop biosensors for various targets such as DNA [129,130,131,132], RNA [133,134], viruses, bacteria, proteins, or small molecules [135]. Some of the molecular probes, such as antibodies and aptamers, can recognize the unique molecular signature of cancer cells; nevertheless, they have relatively weak binding affinities which results in poor signalling and hinders cell targeting. Huang *et al.* [136] used Au-Ag nanorods as a nanoplatform for multivalent binding of up to 80 fluorophore-labeled aptamer probes. This resulted in a much stronger fluorescence signal. As determined by flow cytometric measurements, an enhancement in fluorescence signal in excess of 300-folds was obtained for the nanorod-aptamer-labeled cells when compared with those labelled by individual aptamer probes. Similarly, binding affinity with cancer cells was improved by at least 26-fold through simultaneous multivalent interactions with the cell membrane receptors. Thus, the ability to perform cellular bioimaging and targeting is greatly improved.

**Figure 6:**
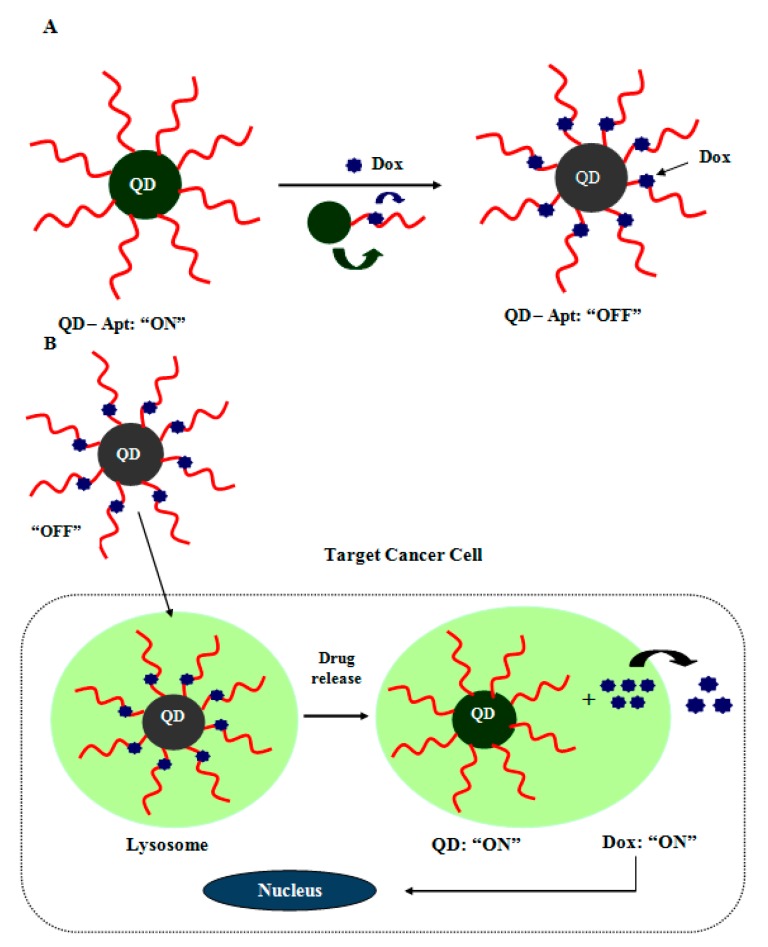
QD-aptamer conjugate serving as both a drug delivery vehicle and a fluorescence imaging agent.

In addition to the above discussed molecular diagnostics, aptamer-nanomaterial conjugates have also been used both as a drug delivery vehicle and fluorescence imager [137]. For example, a QD aptamer conjugate is composed of three components: Prostate cancer cell specific RNA aptamer, QD and the commonly used antitumor drug, doxorubicin (DOX) (Figure 6). Doxorubicin is an anthracycline drug which fluoresces upon intercalating into CG pairs of double stranded oligonucleotide. To form a QD-aptamer-DOX system, aptamers were conjugated to QDs and DOX was intercalated to the aptamer strand [138,139]. This system was initially “off” since the fluorescence of QD was transferred to DOX and the fluorescence of DOX was quenched by the ds RNA aptamer, Figure 6A. Upon injection into cancer cell, DOX is gradually released from the QD-aptamer-DOX system once the target molecule binds onto RNA aptamer. This DOX release recovered the fluorescence of the QD, Figure 6B. Thus the QD-aptamer-DOX system enables monitoring drug release besides allowing targeted drug delivery and bioimaging of the target cells. A similar approach has been applied to super magnetic iron oxide nanoparticles for bioimaging and treatment of prostate cancer [140]. The same methodology could be extended to bioimaging other types of cancer.

Fluorophores, such as quantum dots, emit fluorescence after at excitation at a certain wavelength. Thus, fluorescence-based sensors require a fluorescence microscope or other types of involved fluorimeter for detection. For on-site use and real time, however, it is more practical if detection is carried out without any equipment. Colorimetric detection provides an advantage in this respect, since it allows detection to be made by simple equipment or even by the naked eye. A more cost effective method requiring simple or no instrumentation with high sensitivity and accuracy would be ideal for point-of-care diagnosis. Medley *et al.* [141] developed a colorimetric assay for the direct and sensitive detection of cancer cells. The assay uses aptamer-conjugated gold nanoparticles to combine the spectroscopic advantages of gold nanoparticles and the selectivity and affinity of aptamers. The assay was able to differentiate between different types of control and target cells based on the aptamer used in the assay. The assay also showed excellent sensitivity based on both absorbance measurements and observation with naked eye.

A different approach towards cancer detection which shows great potential in the domain of cancer detection, is based on light-addressable label-free, potentiometric sensor (LAPS) that is coupled to a phage recognition element. The phage-LAPS were able to detect the cancer cells (MDA/MB231 breast cancer cell line) and the cancer biomarker hPRL-3 with high sensitivity [142].

A novel approach towards cancer cell bioimaging could be applied by utilizing the DNA-based biosensor developed by Ohmichi *et al.* [143] for monitoring pH in living cells and *in vitro*. They reported that a combination of two DNA oligonucleotides, 5′AGAAAGAGAAGA-3′ and 5′TCTTTCTCTTCT-3′ demonstrate a new type of structural transition from a Watson-Crick antiparallel duplex to a parallel Hoogsteen duplex as the pH changes form pH 7.0 to 5.0. By labeling this DNA for fluorescence energy transfer, they were able to develop a sensitive pH sensor that could detect changes between pH 5.0 and 7.0. We expect if the same two DNA nucleotides got attached to polyethylene glycol and a suitable targeting agent, a comparable fluorescence change could be observed in many cancerous tissues due to their characteristic acidic pH, thus, allowing their bioimaging.

## 9. Nanobiosensing of Toxicity

Aptamer-conjugated gold nanoparticles (Au NPs) have proved sensitive and specific for the determination of heavy metal ions such as Hg^2+^. Two different Au NPs each functionalized with different thiolated-DNA sequences (5′-thiol-C_10_A_10_TA_10_ and 5′-thiol-C_10_T_10_TT_10_), which are complementary except for a single Thymine-Thymine (T-T) mismatch, have been used. The Au NPs maintained aggregation at higher temperature (T < Tm) in the presence of Hg^2+^ ions that have been known to coordinate selectively at T-T pair [144,145]. Measurement of the changes in Tm was a relatively simple procedure for determining Hg^2+^ concentration because each increase in concentration of 1 µM Hg^2+^ resulted in an increase in Tm by about 5 °C. This biosensing system provided an LOD of 100 nm for Hg^2+^ ions and promises to be valuable in assessing toxicity with heavy metals.

Metal ions, such as lead, can be monitored by using biosensors that combine the high selectivity of catalytic DNA with the high sensitivity of fluorescent detection [146]. Because DNA is stable, cost effective and easily adaptable to optical fibers and microarray technology for device manufacture, this approach holds great promise for on-site and real time quantitative monitoring of metal ions over a wide concentration range in a variety of fields including clinical toxicology. Table 2 summarizes commercially available DNA sensors and commercially available platforms.

**Table 2:** Commercially available DNA sensors and microarrays platforms.

Company Name	Platform	Detection Mechanism	Website
**Nanogen**	Nanochip	Electrochemical	http://www.nanogen.com
**Affymetrix, Santa Clara, CA, USA**	GeneChip^®^ technology either for whole genome or subset gene analysis	Fluorescence	http://www.affymetrix.com
**ADIAGENE, Bruz, France**	Kits for PCR technique (Polymerase Chain Reaction and DNA testing technology for diagnosis of animal diseases	fluorescent probes technology	http://www.adiagene.com
**Agendia, Amsterdam, Netherland**	Genomics platform for tumor gene expression profiling and microarray assay tests that can determine whether an individual patient is at high or low risk for breast or colon cancers recurrence, helping physicians more accurately tailor cancer treatments.	Fluorescence	http://www.agendia.com
**Agilent Technologies, Santa Clara, CA, USA**	1- DNA 500 LabChip^®^ kit provides sizing and quantitation of dsDNA fragments ranging from 25–500 bp.	Fluorescence	http://www.agilent.com
	2- Dual-mode gene expressionmicroarray platform providing one- and two-color gene expression capabilities.		
	3- 2100 Bioanalyzer is a microfluidics-(electrophoresis and flow cytometry) based platform for the analysis of DNA, RNA, proteins and cells.		
**Beckman Coulter Genomics**	DNA variation analysis, whole exome, gene genotyping using next generation sequencing and targeted or individual SNP genotyping using real-time PCR or Sanger sequencing.	Fluorescence	http://www.beckmangenomics.com
**Celera Group, Rockville, MD, USA**	Genetic diagnostic test that are used to detect, characterize, monitor and select treatment for disease,	Fluorescence	http://www.celera.com
**CLONDIAG Chip Technologies, Jena, Germany**	Genetic *in vitro* diagnostics at the point-of-care and in the laboratory based using array tube or arraystrip	Optical	http://www.clondiag.com
**Roche NimbleGen, Madison, WI, USA**	CGH, ChIP-chip, DNA Methylation, AccuSNP, CGS, and Gene Expression microarrays	Optical	http://www.nimblegen.com
**CombiMatrix Diagnostics, USA**	CombiMatrix 12K ElectraSense^®^ microarray offer DNA-based genomic testing services in the areas of (1) Prenatal and Pediatric developmental disorders and (2) Oncology	CMOS	http://www.combimatrix.com
**CustomArray, Inc. Bothell, WA, USA**	ElectraSense microarray; Arrays can be synthesized automatically on the instrument using either the 4 × 2 k™, 12 k™, or 90 k™ array chips. In situ synthesis on up to 32 arrays (for 4 × 2 k format) or 8 arrays (for other formats)	electrochemical	http://customarrayinc.com
**Illumina, San Diego, CA**	Bar coded microbeads	Fluorescence	http://www.illumina.com
**Arrayit Corporation, Sunnyvale, CA, USA**	Arrayit VIP™ (Variation Identification Platform™) technology Universal microarray analysis platform for nucleic acid-based genetic screening, testing, diagnostics, genotyping and single nucleotide polymorphism (SNP) analysis.	Fluorescence	http://www.arrayit.com
**Applied Biosystems, Foster City, CA, USA**	Expression Array System; Microarray assays based on a chemiluminescent detection	chemiluminescent	wwwappliedbiosystems.com
**DNAmicroarray, Inc.**	pre-spotted high density DNAmicroarrays		http://www.dnamicroarray.com
**Eppendorf Biochip Systems, Hamburg, Germany**	Offer microarray solutions for routine applications, including DNAmicroarrays for gene expression analysis, detection of infectious agents, GMOs in food and feed and miRNA analysis.		http://www.eppendorf-biochip.com
**Genisphere LLC, Hatfield, PA, USA**	3DNA™ microarray detection kits include the Array 350™ Kit—an indirect labeling system for cDNA and oligo arrays, the Array 350RP™	Fluorescence	http://genisphere.com
**Infineon Technologies, Munich, Germany**	CMOS based platform	CMOS-based DNA sensor chips with fully electronic readout	http://www.infineon.com
**DNA Electronics Ltd, London**, **UK**	Genalysis^®^	ion-sensitive field effect transistors (ISFETs) based	http://dnae.co.uk
**Oxford Nanopore Technologies®**	developed the GridION™ system and miniaturisedMinION™ devices for electronic single molecule sensing	nanopores to analyse single molecules including DNA/RNA and proteins	http://www.nanoporetech.com

## 10. Conclusions/Outlook

New DNA-based nanobiosensors are being developed using new methods for nanopatterning of new materials. The most important clinical applications of currently available DNA-based nanobiosensors are in the areas of detection of infectious microorganisms, cancer diagnosis and biomarker discovery. The main goal for further development includes development of improved diagnostics that achieve very high sensitivity through the employment of nanomaterials and nanoscale processes that assess markers of specific diseases at points of care. Nevertheless, the development of such platforms is hindered sometimes by the lack of detailed knowledge about specific biochemical interactions, appropriate amplification methods and adequate affinity agents and validated markers. To overcome these deficiencies, it will be necessary to achieve high specificity and sensitivity in nanoscale operations, coupled with a more comprehensive knowledge of the bio-nanointerface affecting nanomaterials target analytes and probes. To improve surface functionality for example, modification processes of nanomaterial should be designed to magnify specific biomolecular interactions and minimize the non-specific ones. One approach is to develop multiple integrated nanobiosensor systems that use besides DNA other nanomaterials such as enzymes, polymers and doped oxides or other components to give the nanobiosensor a very high efficiency and specificity. Such integrated biosensor systems should include all of the sensing components such as software, reagents, and plumbing for nanoscale fluid volumes along with sample processing. Advances in bio nanotechnology and a better understanding of the nano-bio interface will provide robust and multiplexed diagnostic assays where specific signals generated from only a few target molecules can be readily measured with high sensitivity. Advances in bionanotechnology, nanomedicine and nanodiagnostics have largely been the result of persistent collaborations that hybridize the expertise of skillful chemists, biologists, physicians and others with interrelated specialties. Such collaborations should be further encouraged for the sake of achieving more development in the synthesis and characterization of nanomaterial. The value of the advances in nanodiagnostics of the past 10 years and the promise it has for the future of nanomedicine and health care, should not be underestimated. The development of measurement devices based on DNA-based nanobiosensors, which can make hundreds and even thousands of measurements more rapid and cost effective, will become available within the next decade. Further trends in diagnostics will continue in miniaturization of biochip technology to the nanoscale range. Furthermore in the next decade DNA-based nanobiosensors promise to push forward the frontiers of current molecular diagnostics and enable point-of-care diagnosis, integration of nanobiosensors with therapeutics and consequently development of personalized medicine.
